# Dual-responsive (pH/temperature) Pluronic F-127 hydrogel drug delivery system for textile-based transdermal therapy

**DOI:** 10.1038/s41598-019-48254-6

**Published:** 2019-08-12

**Authors:** Sudipta Chatterjee, Patrick Chi-leung Hui, Chi-wai Kan, Wenyi Wang

**Affiliations:** Institute of Textiles and Clothing, The Hong Kong Polytechnic University, Hung Hom, Hong Kong

**Keywords:** Biochemistry, Biological techniques

## Abstract

A dual-responsive hydrogel (pH/temperature) was developed from a thermos-responsive polymer, pluronic F-127 (PF127), and pH-responsive polymers, *N,N,N*-trimethyl chitosan (TMC) and polyethylene glycolated hyaluronic acid (PEG-HA). Gallic acid, the principal component of the traditional Chinese drug Cortex Moutan was loaded into the hydrogel (PF127/TMC/PEG-HA) for possible application in textile-based transdermal therapy as Cortex Moutan has been proven to be an effective drug for the treatment of atopic dermatitis (AD). TMC and PEG-HA were synthesized, characterized (^1^H-NMR and FTIR), and added to the formulations to enhance drug release from the hydrogels, and increase the drug targeting of the carriers. The thermo-responsive properties of the hydrogel were assessed by dynamic viscosity analysis and the tube inversion method, and the pH-responsiveness of the formulation was determined by changing the pH of the external media. Rheology study of the hydrogels showed that complex viscosity and storage/loss moduli for PF127/TMC/PEG-HA hydrogel formulation are higher than PF127 hydrogel. The microstructure analysis by reflection SAXS indicated similar type of frozen inhomogeneity of hydrogel formulations. Various characterizations such as FTIR, SEM, TEM, zeta potential, and degradation of the hydrogel formulation indicated that the PF127/TMC/PEG-HA hydrogel showed better physico-chemical properties and morphology than did the PF127 hydrogel, and drug release was also higher for the PF127/TMC/PEG-HA hydrogel than for PF127. The drug release from hydrogels followed more closely first-order rate model than other rate models.

## Introduction

Textile based transdermal therapy using hydrogel drug delivery systems has been gaining attention for the last few years because of its dual functionality to simultaneously supply moisture and loaded drug onto infected sites on the skin^1–3^. Atopic dermatitis (AD) is the most common skin disease caused by *Staphylococcus aureus* infection. There are several methods to fight against this infection, namely, various corticosteroids, acupuncture, intravenous and hypodermic injections, and herbal therapy^4,5^ but all the conventional methods have some limitations^6^. The disadvantages and limitations related to conventional treatment methods for AD encourage further searches for developing improvised techniques^7^. Functionalized textiles that are coated with metal nanoparticles such as silver or zinc oxide having antimicrobial properties are reported to be effective against AD^8–10^, but long-term exposure of skin to these nanoparticles can produce some toxic effects^11^. Additionally, this method causes the skin to be dehydrated^1^. In this context, textile based transdermal therapy is an effective and affordable skin care treatment for AD because hydrogel systems loaded with drugs and coated on textiles can effectively fight against pathogens providing both moisture and drugs to infected sites on the skin^12,13^. Nevertheless, the formulations of hydrogel-based drug carriers in textile based transdermal therapy play a vital role in the treatment of AD because these carriers need to overcome the skin barrier and target the drug to the designated location^14,15^.

Hydrogels made of hydrophilic polymers are three-dimensional polymeric networks capable of holding a large amount of water^16^. Because, their water-rich structures are similar to biological tissues, hydrogels, especially those with biomimetic applications, are widely applied for developing scaffolds for artificial tissues and organs^17,18^. The biomedical applications of hydrogels have proven to be clinically significant as easy modifications of various physico-chemical properties such as structure, composition, biodegradability, rheological properties, thermal and pH stabilities^19,20^. Hydrogels have become important drug carriers, especially when the hydrogels are made of thermore-sponsive polymers that are capable of showing sol-gel transitions with changes in the external environmental temperature^21–23^. Some synthetic polymers such as poly(*N*-isopropylacrylamide) (pNIPAAm), pluronics^®^ or poloxamers mainly Pluronic F-127 (PF127) are capable of showing sol-gel transition near the body temperature of 37 °C and the unique thermos-responsive property of these polymers is directed towards a wide area of drug delivery applications^21,24^. pNIPAAm, a synthetic thermos-responsive polymer made of NIPAAm monomers by controlled radical polymerization shows a lower critical solution temperature (LCST) within the range of 36.5–37.5 °C, and in the form of injectable hydrogels, pNIPAAm has applications in tissue engineering, and, cell and drug delivery^25^. pNIPAAm-based amphiphilic co-network systems, consisting of both hydrophilic and hydrophobic components covalently bonded in macromolecular structure show tremendous drug delivery applications due to their good biocompatibility, high mechanical strength, solvent nature independent swelling, and nanophase-separated structure^26^. PF127 is a non-ionic triblock copolymer, namely, poly(ethylene oxide)-poly(propylene oxide)-poly(ethylene oxide) (PEO-PPO-PEO) is developed from the self-assembly of two monomeric units ethylene oxide and propylene oxide in water and is capable of showing amphiphilic characters in aqueous environment^12,27^. The LCST of PF127 can be varied from 25 to 37 °C by the changing concentration of PF127 in the formulation, and various modifications are applied to PF127 backbone for developing injectable PF127 hydrogels^27^. PF127 is reported to be non-toxic and biocompatible, and PF127-based thermos-responsive hydrogels are applied as drug carriers for the treatment of different forms of cancer and skin disease^1,28–30^. Some natural polymers such as cellulose, gelatin/collagen, xyloglucan, chitosan, starch, xanthan gum, carrageenans, hyaluronic acid, and dextran are also capable of showing thermo-responsive properties, and thermo-responsive composite hydrogels made of synthetic and natural polymers are reported to perform well as drug carriers^12^. Stimuli-responsive hydrogels are capable of showing changes in their properties in response to changes in the pH of the external environment and pH-responsive hydrogels are made of polymers containing pendant acidic or basic groups that either accept or donate protons in response to the pH change of the external environment^31^. pH-responsive polymers are either cationic [chitosan, poly(dimethylaminoethyl methacrylate), poly(diethylaminoethyl methacrylate)] or anionic (alginate, polyacrylic acid, albumin, hyaluronic acid) and the hydrogels made of these pH-responsive polymers are clinically significant for drug delivery applications^32–35^. Currently, the stimuli-responsive hydrogel systems combined with pH-responsive and thermo-responsive polymers are gaining importance for their suitable applications in drug delivery^36,37^. Dual responsive (pH/temperature) hydrogels made of pH responsive chitosan and thermo-responsive pNIPAAm have already been reported to be an effective drug delivery system for pilocarpine hydrochloride^38^.

A thermo-responsive hydrogel made of PF127 and *N*,*N*,*N*-trimethyl chitosan was applied as a drug delivery system for the anticancer drug docetaxel, and the hydrogel showed a good porous structure with an improved interconnected hydrogel network after modification with TMC^39^. Despite the lack of change in the rheological properties of the hydrogel, drug release from the hydrogel was improved after TMC modification, and the hydrogel was applied for an *in vivo* study to deliver docetaxel to brain tumor in mice^39^. A thermo-responsive hydrogel made of PF127 and hyaluronic acid (HA) was used as a scaffold for tissue engineering and depending on the amount of HA in the final formulation, the LCST of the composite hydrogel varied^40^. In this study, dual-responsive (pH/temperature) hydrogel was developed from the thermo-responsive synthetic polymer PF127 and pH-responsive polymers *N,N,N*-trimethyl chitosan (TMC) and polyethylene glycolated hyaluronic acid (PEG-HA). The dual-responsive hydrogel was applied as a drug carrier for gallic acid, which is the principal component of traditional Chinese drug, Cortex Moutan. In this context, gallic acid was loaded into the hydrogel as an active marker of Cortex Moutan to simplify and understand the release behavior of the hydrogel^1^. The possible application of the gallic acid-loaded hydrogel system (PF127/TMC/PEG-HA) was focused on textile-based transdermal therapy as Cortex Moutan was proven to be an effective drug for the treatment of eczema. A pH-responsive biopolymer with good skin-compatibility, hyaluronic acid (HA)^41^ was derivatized to polyethylene glycolated HA (PEG-HA) by carbodiimide chemistry to increase the drug release from the hydrogels and enhance the percutaneous interaction of drug carriers with the infected sites on the skin^42^. The amine group of polycationic biopolymer chitosan, a pH-responsive polymer^43,44^, was quaternized to TMC by one step methylation process and this compound was added to the formulation to improve the cross-linking network of the hydrogel which can serve as a good drug delivery carrier in textile based transdermal treatment. TMC in drug carrier formulations has been found to be effective for targeted delivery of drugs especially for nasal^45^ and brain^46^ delivery. The morphology and physico-chemical properties of dual-responsive (pH/temperature) hydrogel made of PF127, TMC, and PEG-HA (PF127/TMC/PEG-HA hydrogel) and the drug (gallic acid) release properties of hydrogel for were studied here to develop a suitable drug carrier for the treatment of AD via textile -ased transdermal therapy.

## Materials and Methods

### Materials

Chitosan (low molecular weight), hyaluronic acid sodium salt from *Streptococcus equi* (molecular weight 8000–15000), pluronic F-127, methoxy-polyethylene glycol amine, gallic acid, *N*-(3-dimethylaminopropyl)-*N*′-ethylcarbodiimide hydrochloride, *N*-hydroxysuccinimide, methoxypolyethylene glycol amine, sodium iodide, 1-methyl-2-pyrrolidinone, iodomethane solution (methyl iodide), phosphotungstic acid hydrate, sodium hydroxide, and deuterium oxide were purchased from Sigma-Aldrich Chemical Co. (St. Louis, MO, USA).

### Synthesis of PEG-HA

**Figure 1:**
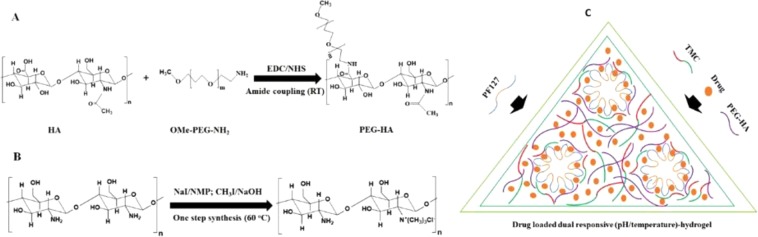
Scheme for the chemical synthesis of PEG-HA from HA (**A**); TMC from chitosan (**B**); the schematic representation of formation of dual responsive (pH/temperature) hydrogel with drug loaded into it (**C**).

PEG-HA was prepared from hyaluronic acid (HA) and methoxy-polyethylene glycol amine (OMe-PEG-NH_2_) using carbodiimide chemistry^47^. HA (0.01 g) dissolved in 5 ml deionized water (pH 6.6) was first reacted with 0.01 g *N*-(3-dimethylaminopropyl)-*N*′-ethylcarbodiimide hydrochloride (EDC) and 0.005 g *N*-hydroxysuccinimide (NHS) for 1 h, followed by reaction with 0.01 g methoxypolyethylene glycol amine (*OMe-PEG*_2000_*-NH*_2_) dissolved in 5 ml water at room temperature under stirring conditions for 24 h. The reaction mixture was dialyzed using a 3.5–5.0 kDa cut-off dialysis membrane (Spectrum) for 1 day against a 100 mM NaCl solution and then, de-ionized water for 3 days. A solid sample of PEG-HA was obtained after lyophilization (Alpha 1–4 LD, Christ). The reaction scheme for the preparation of PEG-HA is given in Fig. 1A.

### Synthesis of TMC

TMC was synthesized using one-step methylation process^48^ in which 0.25 g of chitosan and 0.6 g of sodium iodide were first dissolved in 10 ml of 1-methyl-2-pyrrolidinone at 60 °C with stirring for 24 h, and then, reacted with 2 ml of 0.15 (g/ml) aqueous sodium hydroxide solution and 2 ml of methyl iodide at room temperature under stirring for 2 h. The chemical synthesis scheme of preparing TMC from chitosan is given in Fig. 1B. The product was precipitated using ethanol, followed by isolation of the product using centrifugation and the same process was repeated 2–3 times. The product was dissolved in 5 ml of water, to which 30 ml of 1 M HCl in ethanol (96%) was added to exchange iodide for chloride. Water-soluble TMC, as the final product, was obtained in the form of a white solid powder after centrifugation, followed by washing with ethanol and ether, and finally drying in vacuum at 40 °C.

### Preparation of drug-loaded hydrogel formulations and assessment of their thermo-responsive behavior

The hydrogel forming polymers PF127, TMC, and PEG-HA were added to the water and ethanol mixture along with the drug (gallic acid) to obtain a drug-to-polymer weight ratio (w/w) of 1:9 in the final hydrogel formulation of PF127/TMC/PEG-HA. In brief, 1.35 g PF127, 0.008 g TMC, and 0.008 g PEG-HA were added to the de-ionized water (7.74 g) and ethanol (0.55 g) mixture along with 0.15 g gallic acid dissolved in 0.2 g de-ionized water to obtain a hydrogel formulation (PF127/TMC/PEG-HA) of 10.006 g (∼10.0 g) and PF127 was maintained at 13.5 (wt%) of the total formulation weight. In the drug loaded hydrogel formulation made of only PF127 (PF127), the hydrogel formulation was composed of 1.35 g PF127 dissolved in 7.75 g deionized water, 0.55 g ethanol mixture, and 0.15 g gallic acid dissolved in 0.2 g de-ionized water. The final weight of the hydrogel formulation was maintained at 10.0 g, the drug:polymer ratio was kept at 1:9 (w/w), and PF127 was also maintained at 13.5 (wt%) of the total formulation weight in the sample. The hydrogel formulations in the sol phase were vortexed followed by incubation at 4 °C to obtain a clear solution. The thermo-responsiveness of gel formulations was investigated using dynamic viscosity measurements and the tube inversion method after applying heat or changing the temperature^1^. The formation of hydrogels with thermo-responsive properties is schematically presented in Fig. 1C.

### ^1^H-nuclear magnetic resonance (^1^H-NMR) and Fourier transform infrared (FT-IR) spectroscopy analysis

^1^H-NMR of the chemically synthesized TMC and PEG-HA along with the directly procured chitosan and HA was performed in deuterium oxide, 99 atom% D (D_2_O) using a 500 MHz NMR spectrometer (Varian Unity Inova). The FT-IR spectra of the chemically synthesized compounds and freeze dried hydrogel formulations with drugs were obtained using a Nicolet iS50 FT-IR instrument (Thermo Scientific) within the scanning range of 400–4000 cm^−1^.

### Thermo-responsiveness of hydrogel formulations evaluated by dynamic viscosity analysis and tube inversion method

The dynamic viscosity measurements of the formulations PF127/TMC/PEG-HA and PF127 loaded with drug (gallic acid) were carried out using a DVE Brookfield viscometer with a spindle (s05) setting shear rate at 85 s^−1^ (50 rpm). The percent torque (%) displayed on the instrument was maintained within the range of 10–90% during the viscosity measurement of the samples. The temperature was varied from 5 to 50 °C during the measurement to determine the dynamic viscosity values of the hydrogel formulations at different temperatures. The temperature was increased manually at a rate of 4 °C/min over the range of 5–50 °C and the inflection point for the hydrogel formulations was determined from the dynamic viscosity vs temperature plot. The temperature inside the solution was thoroughly measured by dipping the thermometer into the sample system to determine gelation temperature of the samples.

The sol-gel transition of drug loaded formulations PF127/TMC/PEG-HA and PF127 only was determined by the tube inversion method where the flowability of 10 ml formulations in 20 ml capped glass tubes was checked at different temperatures in the range of 5–50 °C with increments of 1 °C/stage inside thermostatic oven (ESCO Isotherm, Forced Convection Laboratory Oven)^49^. The temperature and flowability of the solutions inside the tubes were checked at each stage to determine the temperature at which the sample stopped flowing inside the tube after tube inversion^45^.

### Rheology study

The modular compact rheometer (MCR302, Anton Paar) with TruStrain^TM^ control was used to measure complex viscosity (Pa.s), storage modulus (Pa), and loss modulus (Pa) of drug loaded formulations PF127 and PF127/TMC/PEG-HA. The rheological study was done with 25 mm parallel plates and the values were taken within the temperature range of 5–50 °C at a constant heating rate of 1 °C/min. All the rheological parameters were recorded as the function of temperature and the temperature at which sol-gel conversion started was determined from the inflection point of the graphs.

### pH-responsiveness of hydrogel materials evaluated by the swelling ratio

The freeze-dried hydrogel formulations of PF127/TMC/PEG-HA and PF127 (0.2 g) were allowed to swell separately in 2 ml of 0.1 M acetate buffer (pH 5.4) and 0.1 M PBS (pH 7.4) for 4 h at 30 °C in capped 5 ml glass vials. At different time intervals, the swelled samples were collected, and the adhered liquid was removed using paper towels, finally, the dried samples were weighed to determine the swelling ratio of the freeze dried samples in response to different pH conditions. The samples collected after the mass measurement were dispersed in the same volume of fresh buffer to maintain uniform sink conditions throughout the experiments. The swelling ratio of the hydrogel formulations was determined from Equation (1)^50^.

1 $${\rm{Swelling}}\,{\rm{ratio}}=(\frac{{W}_{t}-{W}_{0}}{{W}_{0}})$$

where *W*_*t*_ is the final mass of the hydrogels after swelling in buffer and *W*_0_ is the initial mass of the freeze-dried hydrogel samples. All experiments were carried out in triplicate, and the average values are reported with ± standard deviations (SD).

### Scanning electron microscopy (SEM) and transmission electron microscopy (TEM) of the formulations

SEM of the freeze-dried hydrogel samples PF127/TMC/PEG-HA and PF127 was performed using a JSM-6490 (Jeol) system. TEM of the formulations in the sol phase was performed using a JEM-2011 (Jeol) and the samples were negatively stained with phosphotungstic acid (1 wt %, pH 7) to determine their micellar morphology.

### Small angle X-ray scattering (SAXS) study of hydrogels

Reflection SAXS was carried out on SmartLab X-ray diffractometer, Rigaku using X-rays with voltage of 45 kV and current of 200 mA. The incident slit and length limiting slit used in the analysis were 0.1 and 10.0 mm, respectively, under the vacuum of 112 mV.

To interpret the scattering intensity (*I*) data of reflection SAXS curve, the scattering wave vector, *q* is used and given by the following equation:

2 $$q=4\pi \,\sin \,\theta /\lambda $$

where 2*θ* is the scattering angle and *λ* = 1.54 Å is the used wave length of X-rays.

### Zeta potential of the formulations

The zeta potential measurements of the formulations PF127/TMC/PEG-HA and PF127 in the sol phase were performed using a ZetaPlus zeta potential analyzer (BIC) over a storage period of 14 days at 30 °C. The samples were diluted 100 times in de-ionized water before the measurements.

### Hydrogel degradation study *in vitro*

The hydrogel formulations PF127/TMC/PEG-HA and PF127 (10 g) made in 20 ml capped glass vials after incubation at 37 °C were added with 5 ml of 0.1 M phosphate buffer saline (PBS) at pH 7.4 and the samples were incubated at 37 °C under mechanical stirring (70 rpm) for different time intervals to check the degradation of hydrogels in this condition for 14 days^51^. After the predetermined incubation time, the upper part of the buffer was collected from the glass vials and the hydrogels remaining in the vials were weighed prior to replenishment with fresh PBS. The degradation of hydrogels in the swelled condition was determined from the initial and final masses of the samples and are represented as the percentage of the remaining mass of hydrogels in PBS due to mechanical stirring. The experiments were performed in triplicate, and the average value of each set of triplicate experiments is represented with SD.

### The drug release of hydrogel formulations *in vitro*

The cumulative drug (gallic acid) release from the hydrogel formulations PF127/TMC/PEG-HA and PF127 in 0.1 M PBS (pH 7.4) was determined using the dialysis bag method^1^. The Hydrogel formulations (1 ml) were sealed in pre-swollen cellulose membrane dialysis bags (3.5–5.0 kDa cut-off, Spectrum) and immersed into 5 ml of 0.1 (M) PBS buffer (pH 7.4) at 37 °C in a water bath shaken at 100 rpm for 5 days. At designated time intervals, 5 ml of the release media was collected for analysis of gallic acid and replaced with an equal volume of fresh PBS to maintain the sink conditions. Gallic acid (GA) released in the PBS media from the hydrogels was measured spectrophotometrically at 265 nm (UH5300, Hitachi). All release experiments were performed in triplicate and the average value of each set of triplicate experiments is presented with SD.

### Statistical analysis

The drug release and hydrogel stability studies formulations were performed in triplicate, and statistical analysis of the data was performed using SigmaPlot 10.0 software to obtain the standard deviation and average of each set of triplicate experiments.

## Results and Discussion

### Characterization of chemically synthesized TMC and PEG-HA

**Figure 2:**
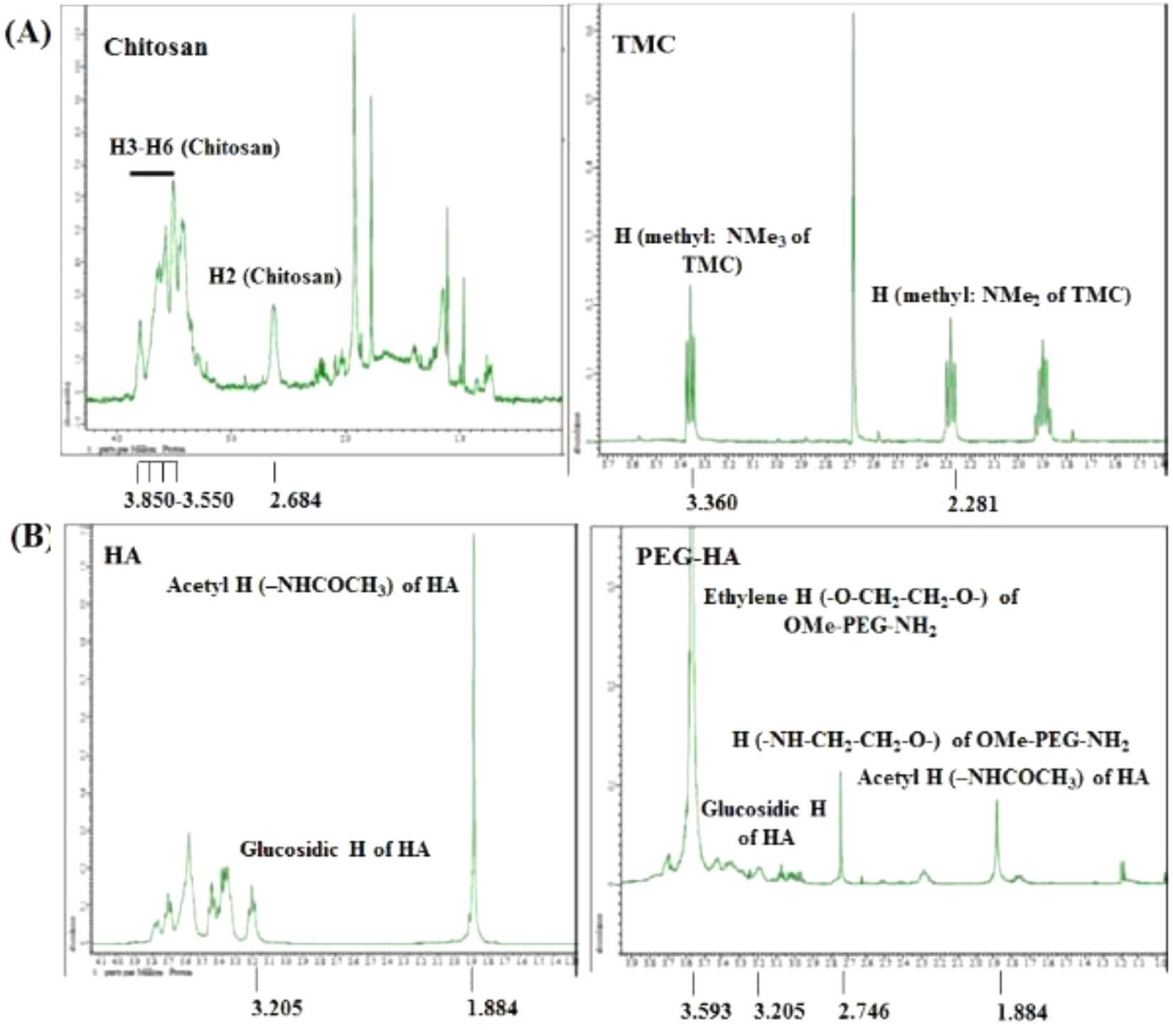
^1^H NMR spectra of chitosan and TMC (**A**); HA and PEG-HA (**B**).

The ^1^H-NMR spectra of TMC and chitosan are shown in Fig. 2A, where chitosan showed peaks at 2.68 ppm for H-2 and multiple peaks in the range of 3.85–3.55 ppm for H-3 to H-6. The peak for the *N*-trimethyl group (-NMe_3_) of TMC was detected at 3.36 ppm confirming the presence of *N*-methylation and referring to quaternized sites. Along with TMC production by one-step *N*-methylation, a modified chitosan derivative with NMe_2_ (2.28 ppm) was synthesized as a side product.

As shown in Fig. 2B, HA showed a peak at 3.21 ppm for glucosidic H and an aeetyl H peak at 1.89 ppm. HA after reaction with OMe-PEG_2000_-NH_2_ showed an ethylene H peak (3.60 ppm) and the (–NH–CH_2_–CH_2_–O–) H peak (2.76 ppm) of OMe-PEG_2000_-NH_2_, indicating that PEG-HA was successfully formed by an amide coupling reaction (Fig. 2B). Other peaks of PEG-HA are very similar to those of HA.

**Figure 3:**
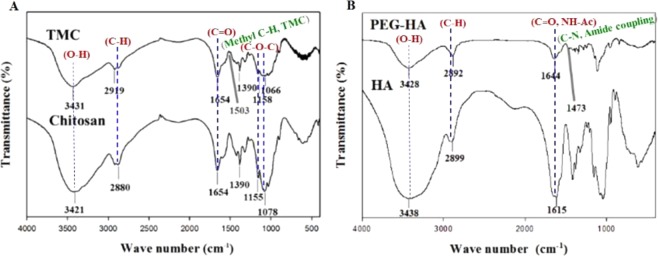
FTIR spectra of chitosan and TMC (**A**); HA and PEG-HA (**B**).

The FT-IR spectra of chitosan and TMC are shown in Fig. 3A. TMC showed characteristic peaks: O–H/N–H stretching (3431 cm^−1^); C–H stretching, pyranose ring (2919 cm^−1^); C=O stretching, amide of NH-Ac (1654 cm^−1^); C–H stretching, methyl of TMC (1503 cm^−1^); C–H bending, CH_3_CO (1390 cm^−1^); and C–O–C stretching (1158, 1066 cm^−1^). Figure 3A shows that the spectral peaks of chitosan are similar to those of TMC. Chitosan showed O–H/N–H stretching at 3421 cm^−1^, C–H stretching of the pyranose ring at 2880 cm^−1^, C=O stretching of the amide of NH-Ac at 1654 cm^−1^, C–H bending of CH_3_CO at 1390 cm^−1^, and C–O–C stretching at 1155 and 1078 cm^−1^. The peak at 1503 cm^−1^ for the C–H bonds of methyl groups in TMC corresponds to the addition of trimethyl groups to the amine groups of chitosan.

The characteristic FTIR peaks of PEG-HA shown in Fig. 3B are O–H stretching (3428 cm^−1^), C–H stretching, pyranose ring (2892 cm^−1^), C=O stretching, NH-Ac (1644 cm^−1^), and C-N stretching (1473 cm^−1^). HA showed similar characteristic peaks to those of PEG-HA and the spectral peaks of HA are O–H stretching (3438 cm^−1^); C–H stretching, pyranose ring (2899 cm^−1^); and C=O stretching, NH-Ac (1615 cm^−1^). The peak found at 1473 cm^−1^ for PEG-HA indicates amide coupling of HA with OMe-PEG_2000_-NH_2_.

### Dynamic viscosity analysis and tube inversion results (thermo-responsiveness of hydrogel formulations)

**Figure 4:**
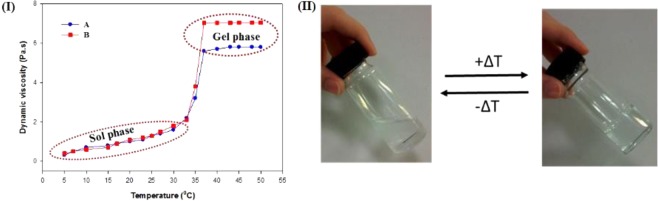
Dynamic viscosity study of drug loaded (**A**) PF127 hydrogel & (**B**) PF127/TMC/PEG-HA hydrogel (I); digital image of sol-gel transition of drug loaded hydrogel (PF127/TMC/PEG-HA) with temperature change via tube-inversion method (II).

In this study, the dynamic viscosity values of the formulations were measured as a function of temperature with a fixed shear rate of 85 s^−1^, and the conversion of the sol phase to the gel phase with temperature is depicted in Fig. 4I. The dynamic viscosity (Pa.s) values of the gallic acid-loaded hydrogel formulations PF127/TMC/PEG-HA and PF127 gradually changed with increaseing temperature in the range of 5–50 °C, and from the point of inflection, both formulations clearly showed a sol-gel transition at 37 °C. Moreover, the PF127content of both formulations was 13.5 wt% of the total weight of hydrogel formulations, and both samples showed a gelation temperature of 37 °C. The dynamic viscosity values of the PF127/TMC/PEG-HA and PF127 hydrogels at 37 °C were 7.02 Pa.s and 5.6 Pa.s, respectively. The higher dynamic viscosity value of PF127/TMC/PEG-HA than that of PF127 at 37 °C was due to the addition of TMC and PEG-HA in the PF127-based hydrogel system and that created enhanced inter-micellar interactions by increasing hydrophobicity of the whole system^1^. Moreover, the higher dynamic viscosity value of the PF127/TMC/PEG-HA hydrogel at the point of gelation suggested better resistance to deformation under stress. In the literature, PF127 is reported to act like a non-Newtonian fluid in the gel phase (37 °C) and the dynamic viscosity values varied as a function of shear rate^52^. PF127 in the sol-phase acted like a Newtonian fluid^52^. In the present study, the dynamic viscosity values of formulations in sol phase at 5 °C were 0.40 Pa.s and 0.32 Pa.s for PF127/TMC/PEG-HA and PF127, respectively, which are in good agreement with previously reported values in the literature^52^.

The Tube inversion method was applied to visualize gelation with temperature change by measuring the flowability of PF127/TMC/PEG-HA [Fig. 4(II)] and the system showed reversible gelation with a sol-gel transition at 37 °C. PF127 chains along with two other compounds (TMC and PEG-HA) use temperature as trigger and form hydrogels through reversible physical linking of the polymer chains^53^. The hydrogels revert to the solution state after the thermal stimulus is removed. The inter-micellar aggregation of thermo-responsive polymers near the gelling temperature shows a positive entropy change (ΔS) and a negative free energy change (ΔG) of aggregation^54^. The water-water associations cause an increase in entropy known as the hydrophobic effect, which is the guiding force for gel formation at LCST^54^.

### Rheology study

**Figure 5:**
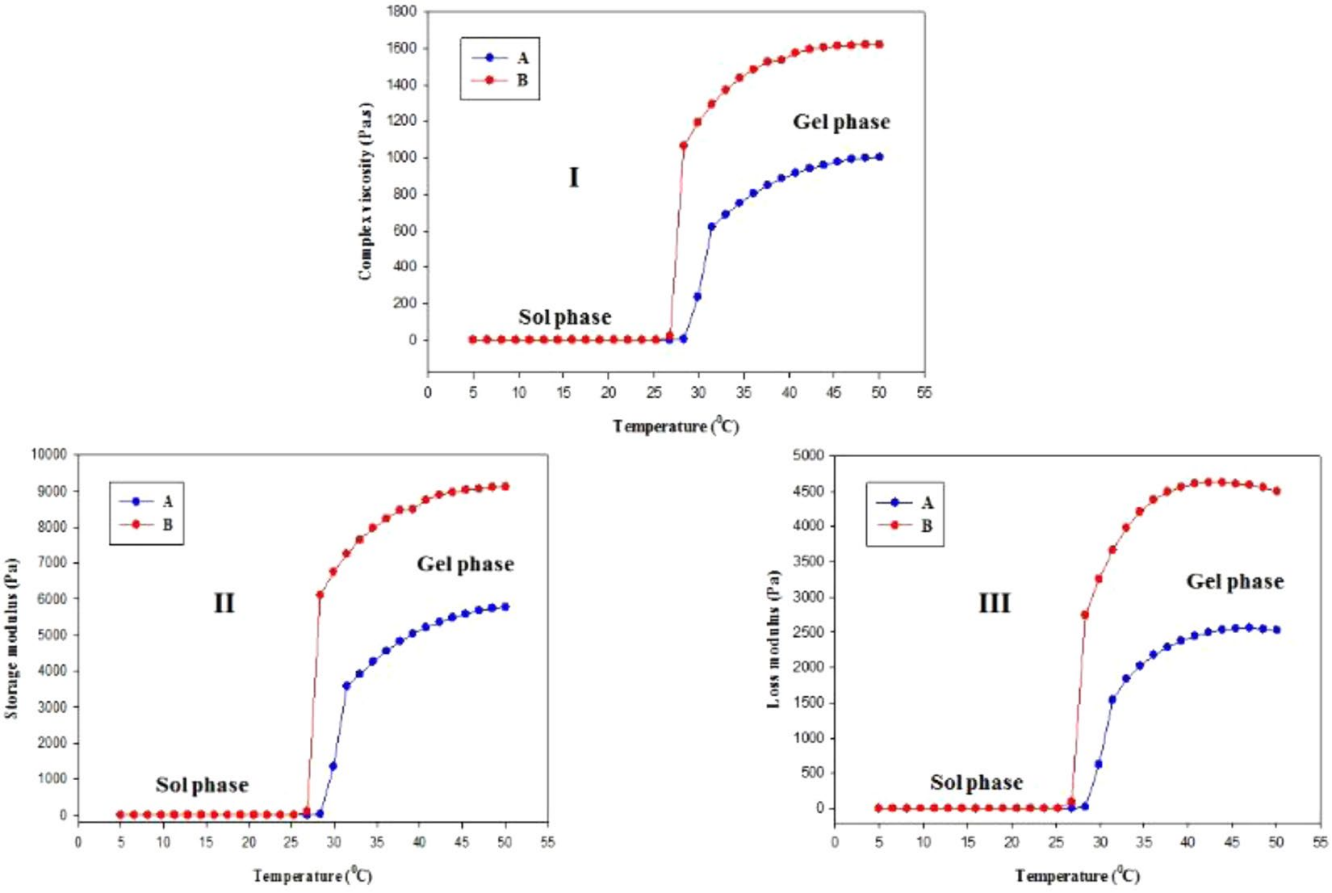
Complex viscosity (I), storage modulus (II), and loss modulus (III) change of drug loaded (**A**) PF127 & (**B**) PF127/TMC/PEG-HA formulations with temperature.

The rheological parameters of the formulations namely, complex viscosity (Fig. 5I), storage modulus (Fig. 5II), and loss modulus (Fig. 5III) have been illustrated as the function of temperature. As shown in Figure 5, all the rheological parameters are highly dependent on temperature and the sol-gel conversion of both formulations has been found to be started near 30 °C as obtained from the inflection point of the graphs. After gel formation, all the rheological parameters of both formulation are found to be much higher than their sol phase. The complex viscosity (I), storage modulus (II), and loss modulus (III) values of PF127/TMC/PEG-HA are significantly higher than those of PF127 in their gel state indicating that the modified PF127 hydrogel with TMC and PEG-HA are mechanically stronger than PF127 hydrogels. The improved rheological parameters of PF127/TMC/PEG-HA hydrogel might occur due to strong inter-micellar interactions and TMC and PEG-HA would possibly enhance the stability of micelles formed by PF127 in the hydrogel.

### Swelling study (pH-responsiveness of hydrogel formulations)

**Figure 6:**
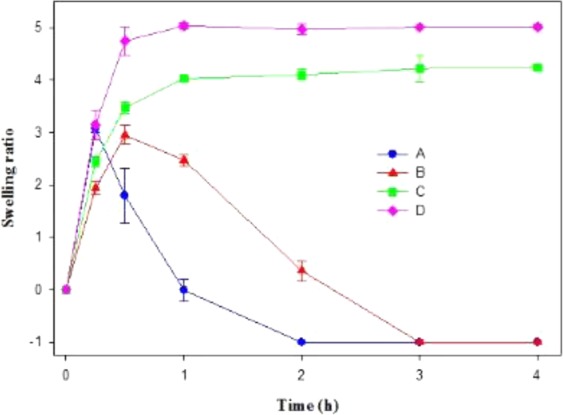
pH-responsiveness of freeze-dried hydrogel materials in 0.1 (M) acetate buffer (pH 5.4) and at 30 °C for drug loaded formulations (**A**) PF127 & (**B**) PF127/TMC/PEG-HA; 0.1 (M) PBS buffer (pH 7.4) and at 30 °C for drug loaded formulations (**C**) PF127 & (**D**) PF127/TMC/PEG-HA. Data represent average of triplicate experiments ± SD.

The swelling of freeze-dried hydrogel particles under acidic pH (pH 5.4) using 0.1 M acetate buffer against time (Fig. 6) at 30 °C revealed that the gel particles formed by PF127/TMC/PEG-HA in the swelled state resisted complete dissolution until 30 min and then, it started to be dissolved in the media to degrade completely just after 2 h. In contrast, PF127 in the swelled state resisted degradation until 15 min, and was completely degraded after 1 h. The hydrophilicity of PF127 was increased under acidic conditions due to the polymer-water interaction, which resulted in rapid dissolution of the gel structure^21^. The pH-responsiveness of the freeze-dried hydrogel particles was modified after adding TMC and PEG-HA in the system, and the enhanced inter-micellar interaction of the modified hydrogel system rendered resistance to the acid mediated degradation/dissolution of gel particles for a longer time.

The swelling of gel particles under neutral pH (7.4) using 0.1 M PBS at 30 °C revealed that the hydrogel particles remained swelled for 4 h without any evidence of dissolution. The swelling ratio of PF127/TMC/PEG-HA (5.01) at pH 7.4 after 4 h was higher than that of PF127 (4.23) indicating that the interconnected porous network of PF127/TMC/PEG-HA accumulated more water molecules in their structure in the swelled state (Fig. 6).

### Hydrogel degradation study under mechanical stirring (mechanical stability test)

**Figure 7:**
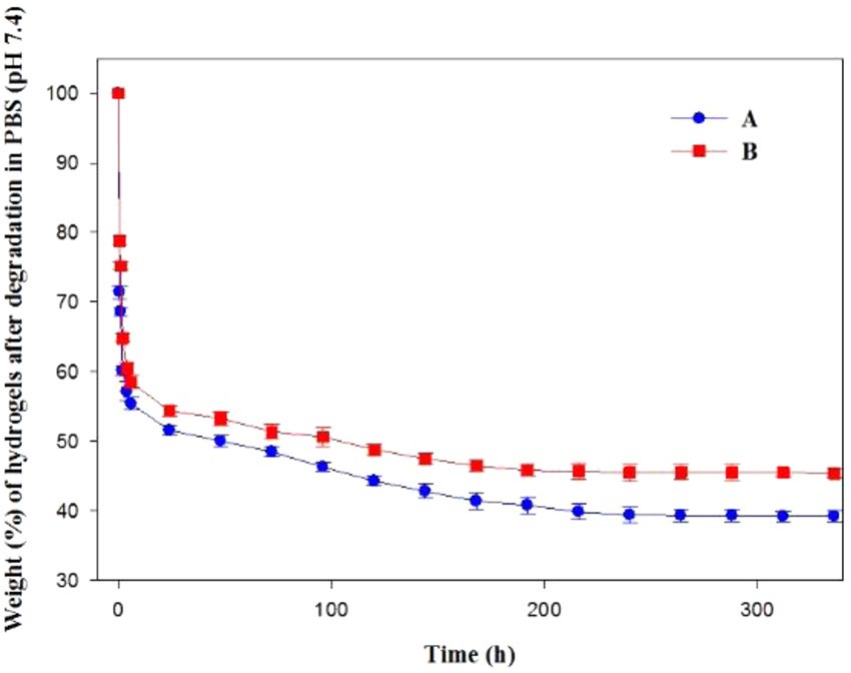
Dissolution study at pH 7.4 and 37 °C in 0.1 (M) PBS buffer for drug loaded dual responsive (pH/temperature) hydrogels (**A**) PF127 & (**B**) PF127/TMC/PEG-HA. Data represent average of triplicate experiments ± SD.

The hydrogel degradation study of PF127/TMC/PEG-HA and PF127 under mechanical stirring (70 rpm) for 14 days in neural pH conditions indicated that PF127/TMC/PEG-HA resisted gel degradation to a higher extent than did the PF127 hydrogel (Fig. 7). The hydrogel made of PF127/TMC/PEG-HA showed a remaining mass of 45.4% after mechanical stirring for 14 days, while the PF127 system showed higher mass loss after 14 days (remaining mass of 39.2%). Therefore, the mechanical stability of the hydrogel was increased after the addition of TMC and PEG-HA to PF127 polymer chains in the formulation because a more interconnected hydrogel structure was formed by this modification.

### SEM and TEM study of hydrogel formulations

**Figure 8:**
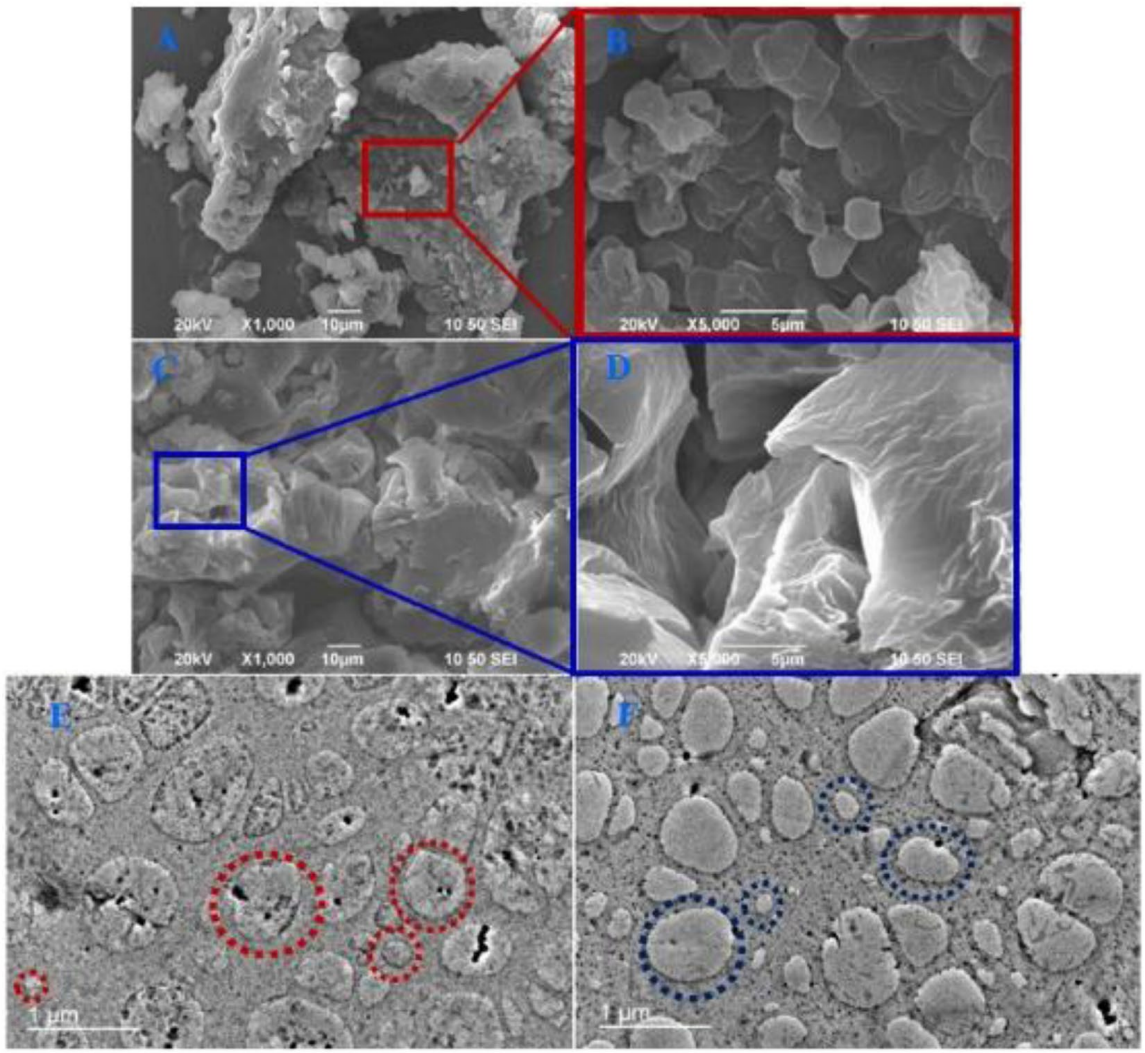
SEM images of drug loaded hydrogels (**A**) X1000 of PF127 & (**B**) magnified (X5000) image of red box in A; (**C**) X1000 of PF127/TMC/PEG-HA & (**D**) magnified (X5000) image of blue box in C; TEM images of drug loaded formulations in sol phase for (**E**) PF127 & (**F**) PF127/TMC/PEG-HA.

The SEM images of gallic acid loaded PF127 (Fig. 8A,B) and PF127/TMC/PEG-HA (Fig. 8C,D) hydrogels in the freeze-dried state show agglomerated porous structures with irregular pore sizes after freeze drying, as the removal of water caused all interconnected networks in the hydrogel to stack. Nevertheless, the image of PF127/TMC/PEG-HA (magnified blue box of Fig. 8C) in Fig. 8D exhibits a better interconnected network with more distinctive pores than that of PF127 alone (magnified red box of Fig. 8A) as inter-micellar interactions of PF127 chains were enhanced after modification with TMC and PEG-HA^55^. The loaded drug inside the hydrogel was relatively more favorably distributed inside the gel structure of PF127/TMC/PEG-HA and the sustained and controlled drug release was better in the modified PF127system due to the more interconnected porous structure in the original formulation.

The TEM images of PF127 (Fig. 8E) and PF127/TMC/PEG-HA (Fig. 8F) formulations in the sol state show micellar aggregates/interconnected micelles varying from 100 to 1000 nm in size which appear on the images as granules of varying shapes as indicated by the red dotted circles in Fig. 8E and the blue dotted circles in Fig. 8F. As shown in Fig. 8, the micelles formed in PF127/TMC/PEG-HA (F) were more compact and stable than those made of PF127 only (E). The micelles were mainly formed by PF127 polymer chains, and TMC and PEG-HA in the PF127/TMC/PEG-HA formulation influenced the formation of stable and compact inter-micellar structures or micellar aggregates of PF127 through the hydrophobic interaction. Therefore, the delivery system made of PF127/TMC/PEG-HA hydrogel could show good drug release due to their stable inter-micellar structures.

### SAXS study of hydrogels

**Figure 9:**
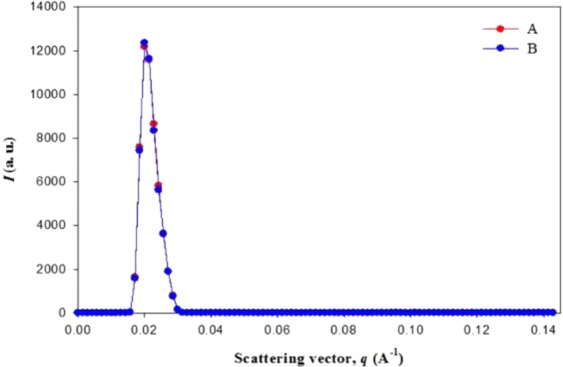
Reflection SAXS by measuring *I* (a. u.) vs q (Å^−1^) of drug loaded (**A**) PF127 and (**B**) PF127/TMC/PEG-HA hydrogels.

The freeze-dried form of the hydrogels coated on the glass plate was placed parallel to the sample holder on the instrument for reflection SAXS. As shown in Fig. 9, the values of *I* (a. u.) of the hydrogel samples were plotted against *q* (Å^−1^). SAXS in reflection mode is done when X-ray hits a flat sample almost parallel to the surface and gives the idea of inhomogeneity of the hydrogel network^56^. The plot of *I* (a. u.) *vs q (*Å^−1^) in Fig. 9 has shown peak appearance at *q* = 0.02 Å^−1^ which is observed in hydrogels indicating the presence of frozen inhomogeneity and this is due to the presence of highly electron density crystallized region originating from the inherent network defects of hydrogels^57^.

### Zeta potential of hydrogel formulations

The zeta potential of the formulations in the sol phase was measured for 14 days to monitor the stability of the components in the system during storage. The surface charge of the components in the formulation determines their stability, solubility and clearance^58^, and the surface charge of a component is measured by the zeta potential. Both PF127 and PF127/TMC/PEG-HA formulations with drug (gallic acid) in the sol phase exhibited negative zeta potential values (Table 1). PF127 in the sol phase with loaded gallic acid showed a negative zeta potential of −18.7 mV ± 6.1 at 30 °C and PF127/TMC/PEG-HA with drug (gallic acid) in the sol phase showed a negative zeta potential value of −16.3 mV ± 5.9. As shown in Table 1, the zeta potential values of PF127 and PF127/TMC/PEG-HA were −18.7 mV ± 6.5 and −14.6 mV ± 7.7, respectively, after 14 days of storage at 30 °C. Therefore, all components in the formulations were uniformly distributed in the system without precipitation as no significant change in zeta potential values was found for both formulations over the storage period. Moreover, the surface charges of the micelles are not changed significantly in PF127/TMC/PEG-HA formulation as TMC and PEG-HA are oppositely charged and therefore, the resultant zeta potential of PF127/TMC/PEG-HA is similar to the zeta potential of formulation with PF127 only.

**Table 1:** Zeta potential data^A^ of drug loaded dual responsive (pH/temperature) hydrogel formulations in sol phase over storage for 14 days.

Storage tome (days)	Zeta potential of PF127 formulation in sol phase (mV)	Zeta potential of PF127/TMC/PEG-HA formulation in sol phase (mV)
0	−18.66 ± 6.06	−16.30 ± 5.88
1	−18.73 ± 7.87	−16.84 ± 2.41
2	−20.51 ± 9.78	−14.46 ± 8.28
3	−18.28 ± 8.00	−15.31 ± 7.83
4	−18.58 ± 7.87	−16.74 ± 6.83
5	−19.81 ± 6.81	−13.20 ± 4.63
6	−21.70 ± 2.85	−15.14 ± 5.18
7	−18.57 ± 5.63	−14.40 ± 6.62
8	−17.40 ± 8.47	−13.65 ± 6.12
9	−19.46 ± 5.96	−14.03 ± 4.37
10	−20.11 ± 7.65	−15.02 ± 6.60
11	−19.14 ± 2.35	−14.98 ± 4.76
12	−18.90 ± 5.62	−13.20 ± 6.61
13	−17.67 ± 7.26	−14.14 ± 2.32
14	−18.74 ± 6.47	−14.56 ± 7.65

^A^Zeta potential data represent average of triplicate experiments ± SD.

### FTIR study of drug loaded hydrogels

**Figure 10:**
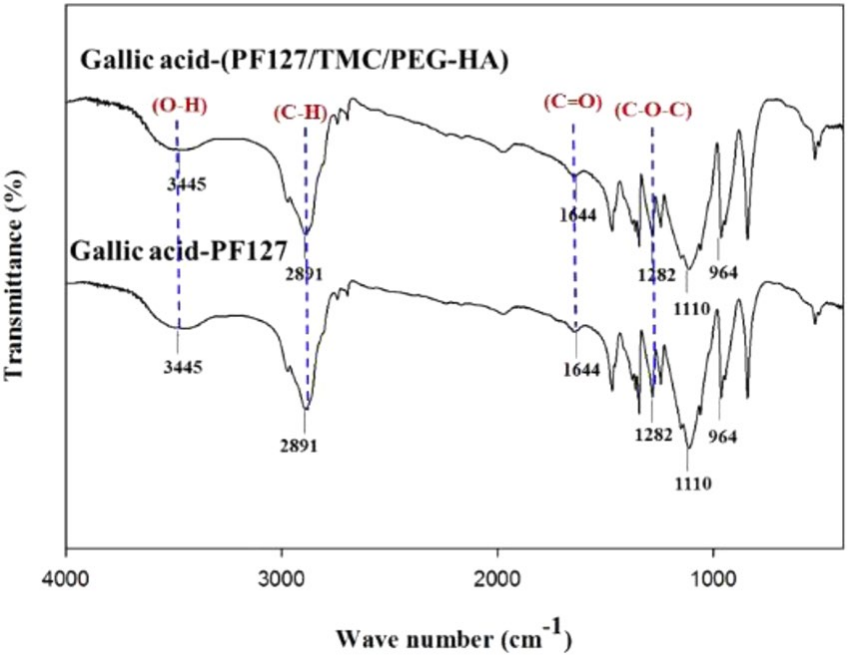
FTIR spectra of drug loaded dual responsive (pH/temperature) PF127 and PF127/TMC/PEG-HA hydrogels.

Figure 10 shows the characteristic FTIR peaks of PF127/TMC/PEG-HA hydrogel loaded with gallic acid in the freeze-dried form (cm^−1^), and these peaks are 3445 (O–H stretching), PF127, TMC, PEG-HA, and gallic acid); 2891 (C–H stretching), PF127, TMC, PEG-HA, and gallic acid; 1644 (C=O stretching), TMC and PEG-HA; 1282 (C–O–C stretching), PF127, TMC, and PEG-HA; 1110 (C-C-O symmetric stretching) PF127; 964 (C-C-O asymmetric stretching), PF127. Similar characteristic peaks were observed from FTIR analysis of PF127 hydrogel loaded with gallic acid. The FTIR peaks of the PF127 hydrogel loaded with gallic acid in the freeze-dried form are 3445 cm^−1^ (O-H stretching), PF127 and gallic acid; 2891 cm^−1^ (C–H stretching), PF127 and gallic acid; 1282 cm^−1^ (C–O–C stretching), PF127; 1110 (C–C–O symmetric stretching) PF127; and 964 (C–C–O asymmetric stretching), PF127. The broad peak found at 3445 cm^−1^ of both hydrogel formulations suggested that gallic acid was effectively loaded into the hydrogels.

### Cumulative release study

**Figure 11:**
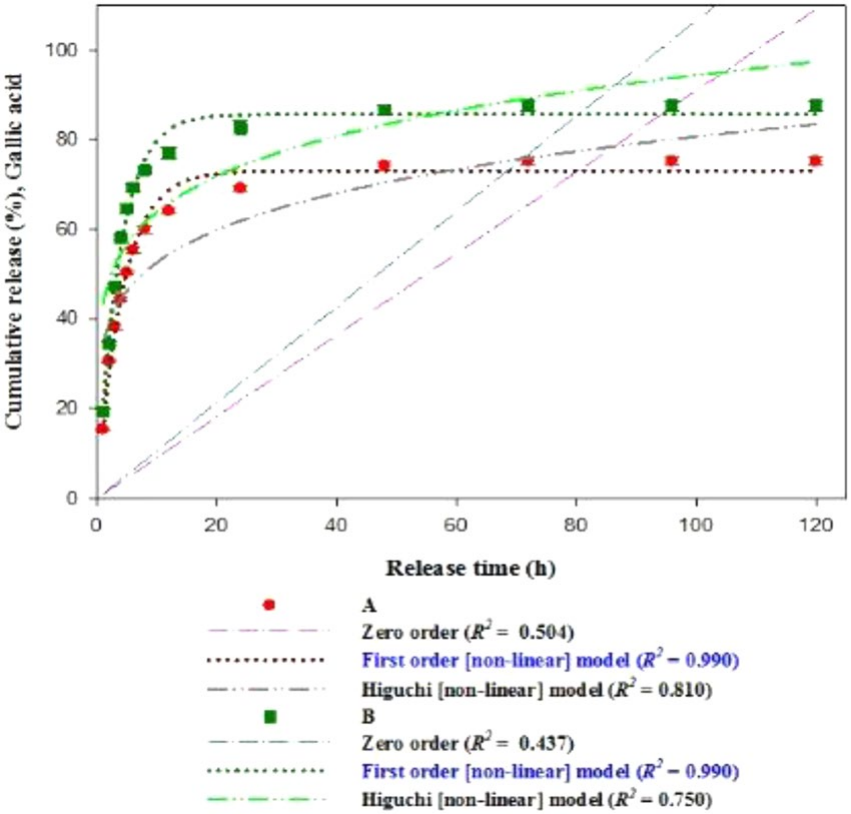
Cumulative release (%) of drug (Gallic acid) from (**A**) PF127 and (**B**) PF127/TMC/PEG-HA hydrogels at pH 7.4 and 37 °C for 5 days in 0.1 (M) PBS buffer. Data represent average of triplicate release experiments ± SD and fitting of drug release data to three different rate models zero-order, first-order and Higuchi.

The release study was performed to examine the release of gallic acid from PF127/TMC/PEG-HA and PF127 hydrogels in 0.1 M PBS (pH 7.4) and at 37 °C (Fig. 11). Both formulations showed a burst release of the drug (gallic acid) at an initial stage (within 5 h) with 64.60% ± 1.112 and 50.31% ± 0.411 drug release for PF127/TMC/PEG-HA and PF127 hydrogels, respectively. After 5 days, a cumulative drug release of 87.61% ± 1.112 and 75.20% ± 0.850 was registered for PF127/TMC/PEG-HA and PF127 hydrogels, respectively, indicating that the modified hydrogel system made of PF127 with TMC and PEG-HA worked better as a drug delivery system. The morphological changes of PF127/TMC/PEG-HA, such as enhanced inter-micellar interactions and a well-formed porous network structure improved the drug release under neutral pH conditions.

Figure 11 The cumulative release values of drug (gallic acid) from the hydrogels are fitted with different kinetic rate models (Fig. 11), and the rate constants of different rate models for all hydrogel varieties are tabulated in Table 2. The fitting of release data to various rate models are expressed in *R*^2^ values (Fig. 11).

**Table 2:** Constants of different rate models for release of drug (gallic acid) from dual-responsive hydrogel.

Drug loaded formulations	Zero-order rate model *K*_0_ (sec^−1^)	First-order rate model *K*_1_ (sec^−1^)	Higuchi rate model *K*_*H*_ (sec^−0.5^)
PF127	2.53 × 10^−4^	6.70 × 10^−5^	5.72 × 10^−1^
PF127/TMC/PEG-HA	2.94 × 10^−4^	7.50 × 10^−5^	7.20 × 10^−1^

The zero-order rate model is given by the equation:

3 $${Q}_{t}={Q}_{0}+{k}_{0}t$$

where *Q*_*t*_ is the cumulative amount of drug release from the hydrogel at time *t* (h), *Q*_*o*_ is the initial amount of drug loaded into the hydrogel, and *k*_0_ is the zero-order rate constant (sec^−1^). The drug release rate by zero-order rate equation is independent of the initial drug amount loaded into the hydrogel.

The first-order rate model is given by the following non-linear form:

4 $${Q}_{t}={Q}_{0}(1-{e}^{-{k}_{1}t})$$

where *Q*_*t*_ is the cumulative amount of drug release from the hydrogel at time *t* (h), *Q*_*o*_ is the initial amount of drug loaded into the hydrogel, and *k*_1_ is the first-order rate constant (sec^−1^). The drug release rate by first-order rate equation is dependent on its concentration (the initial drug amount loaded into the hydrogel).

Higuchi rate equation suggests release of drug from hydrogels by diffusion method and the non-linear form of the Higuchi rate equation is:

5 $${Q}_{t}={k}_{H}{t}^{0.5}$$

where *Q*_*t*_ is the cumulative amount of drug release from the hydrogel at time *t* (h), and *k*_*H*_ is the Higuchi constant (sec^−0.5^).

The fitting of release data to the different rate models (Fig. 11) indicates that drug release rate of the hydrogel formulations closely follows first-order rate equation as both gallic acid loaded PF127 and PF127/TMC/PEG-HA hydrogels show *R*^2^ values of 0.990 which are higher than those obtained from other rate models used in this study. Therefore, the rate of drug release from the hydrogels is dependent on the initial concentration of drug loaded into the hydrogels. As found in Table 2, *k*_1_ (sec^−1^) is lower than other rate constants for both varieties of hydrogels, and so, the sustained release of drug from the hydrogel follows more closely first-order rate model than any other rate model used here.

## Conclusions

A dual-responsive (pH/temperature) hydrogel (PF127/TMC/PEG-HA) was loaded with gallic acid for possible application in textile-based transdermal therapy especially for the treatment of AD. The hydrogel system made of thermo-responsive polymer PF127 was added with two pH-responsive chemically synthesized compounds, namely, TMC form chitosan and PEG-HA from HA, and the synthesis of the compounds was confirmed by ^1^H-NMR and FTIR. The thermo-responsiveness of the hydrogel system was confirmed by a dynamic viscosity study and the tube inversion method, and the PF127/TMC/PEG-HA formulation underwent a sol-gel transformation at 37 °C. The change of complex viscosity and storage/loss moduli of formulations with temperature indicated that the rheological parameters are temperature dependent and sol-gel conversation was started near 30 °C for both varieties. Furthermore, all the rheological parameters are higher for PF127/TMC/PEG-HA than PF127 in the hydrogel state indicating higher mechanical stability of modified PF127 hydrogel system with TMC and PEG-HA. The pH-responsiveness of the freeze-dried hydrogel material indicated complete dissolution in acidic pH (5.4), and considerable stability at pH 7.4. The microstructure analysis by reflection SAXS indicated similar type of frozen inhomogeneity of hydrogel formulations and the frozen inhomogeneity of hydrogels was found at *q* = 0.02 Å^−1^. The FTIR study of the drug-loaded formulation in the freeze dried state indicated that the drug was properly loaded into the gel structure of the formulation and that the release was much higher for the PF127/TMC/PEG-HA hydrogel system than that for the PF127 hydrogel. SEM and TEM of the formulations indicated that the modified hydrogel system showed a good interconnected network and that the micelles were well formed, respectively, which might cause PF127/TMC/PEG-HA to perform better performance of as a drug carrier for gallic acid. The sustained drug release from the hydrogel formulations followed more closely first-order rate model than other rate models.

The main focus of future publications will be to load traditional Chinese medicine in the dual responsive hydrogel system and apply it to textile-based transdermal therapy. The forthcoming research will comprise drug release studies, other physico-chemical characterizations of the hydrogel system, and, most importantly, clinical trials for successful application in the treatment of AD by textile based transdermal therapy.
